# Microbial composition of sweetness-enhanced yoghurt during fermentation and storage

**DOI:** 10.1186/s13568-020-01069-5

**Published:** 2020-07-25

**Authors:** Giuseppina Luzzi, Erik Brinks, Jan Fritsche, Charles M. A. P. Franz

**Affiliations:** 1 Department of Microbiology and Biotechnology, Max Rubner-Institut, Hermann-Weigmann-Str. 1, 24103 Kiel, Germany; 2 Department of Safety and Quality of Milk and Fish Products, Max Rubner-Institut, Hermann-Weigmann-Str. 1, 24103 Kiel, Germany

**Keywords:** Yoghurt, Dairy microbiota, Metagenomics, Fermentation, Reformulation, Sweetness enhancement

## Abstract

The reformulation of dairy products to contain less added sugar can contribute to reducing sugar consumption, thereby reducing the risk of non-communicable diseases. The objective of this study was to investigate the microbial ecology of reformulated yoghurt, which was produced using bi-enzymatic modification of lactose to increase its sweetness by a factor of 2–3. Ultimately, this reformulation strategy could reduce the amount of added sugar needed for equal sweetness of the end product. The bi-enzymatic modification relied on utilisation of a β-galactosidase enzyme to convert the milk sugar lactose to galactose and glucose, followed by the enzymatic conversion of the glucose moiety to fructose using a glucose isomerase. The microbial ecology of reformulated yoghurt produced with two mixed starter culture preparations containing either *Streptococcus* (*S*.) *thermophilus* and *Lactobacillus* (*Lb*.) *delbrueckii or S. thermophilus*, *Lb. acidophilus* and *Bifidobacterium* sp. strains, was analysed during fermentation and cool storage using 16S rRNA based metagenomics. None of the yoghurt samples showed a significant difference in microbial composition between sweetness-enhanced and regular milk at all sampling time points during manufacture and storage of yoghurt. However, a significant difference between the microbiota of inoculated milk before and after fermentation was observed. In both types of yoghurt, the starter culture genera dominated the microbial ecology at the end of fermentation as expected, reducing the possibility of growth of potentially pathogenic or spoilage bacteria possibly resulting from a changed carbohydrate spectrum.

## Introduction

Sugar is vital for the healthy functioning of the human body, yet its excess consumption should be minimised in order to reduce the risk of non-communicable diseases such as diabetes mellitus type II. In Germany, dairy products contribute to the daily added sugar consumption by 7–9% (Alexy et al. 2003; Max Rubner-Institut 2008; Max Rubner-Institut 2016). Hence, the reformulation of fermented dairy products to contain less added sugar can contribute to reducing sugar intake in the population.

Yoghurt and sweetened dairy products can contain up to 22% (w/w) total sugar (Max Rubner-Institut 2016). The natural milk sugar lactose is present in these products at about 4–6%, however, this sugar is not perceived as sweet (Chandan 2006). One approach to reformulate sweetened dairy products is to increase the sweetening power of lactose by converting this disaccharide into its sweeter monosaccharide components using a β-galactosidase enzyme to yield galactose and glucose (Harju et al. 2012). A further enzymatic process can then be applied, in a bi-enzymatic process proposed by Lorenzen et al. (2013), to convert roughly half of this glucose into the even sweeter sugar fructose, through implementation of a glucose isomerase enzyme. As the individual monosaccharide components are sweeter than lactose, the overall sweetness of lactose can be increased by a factor of 2–3.

Enzymatically sweetened milk can be used for the production of dairy products to increase the overall sweetness, so that products such as flavoured yoghurt or pudding can be manufactured with less added sugar when compared to the original product. In a technological and sensory study run in parallel to the current study, Luzzi et al. (2020) demonstrated that a 10–20% reduction in added sugar was possible in yoghurt and pudding samples when applying a bi-enzymatic conversion of lactose, whilst retaining comparable sensory characteristics of the final product. Luzzi et al. (2020) compared the microbial growth and acidification progression of commercial yoghurt starter culture bacteria in enzymatically-sweetened milk to that in regular milk using traditional, culture-dependent methods. It was found that starter bacteria behaved similarly in both the sweetness-enhanced and the regular milk, with starter *Streptococcus* (*S.*) sp. strains growing rapidly to 10^9^ cfu/mL and starter *Lactobacillus* (*Lb*.) sp. strains growing to 10^6^ cfu/mL during fermentation in both a traditional and a probiotic yoghurt culture, thus effectively reducing the pH to ca. 4.5 in both sweetness-enhanced and regular yoghurt. The bacterial count of *Bifidobacterium* species in the probiotic yoghurt after fermentation was determined to be ca. 10^6^ cfu/mL.

Considering the comparable fermentation properties of two different yoghurt cultures in reformulated, sweetness-enhanced milk compared with regular milk as demonstrated by Luzzi et al. (2020), the current study analysed the exact microbial ecology of the same reformulated yoghurt samples using 16S rRNA metagenomic analyses. The aim of this study therefore was to determine whether the changes in the fermentable sugar spectrum would affect the microbial composition of the yoghurt fermentation with respect to the starter cultures or milk autochthonous microbiota.

## Materials and methods

### Preparation of sweetness-enhanced yoghurt and sampling

Raw cow’s milk for all experiments was obtained from the experimental farm (Schädtbek, Germany) of the Max Rubner-Institut in Kiel where a herd of almost 100 dairy cows is kept. Sweetness-enhanced milk for yoghurt production was prepared using the bi-enzymatic system of lactose conversion described by Lorenzen et al. (2013). In a concurrent study, the carbohydrate composition of the regular and sweetness-enhanced milk samples used for yoghurt production were analysed and reported (Luzzi et al. 2020). Yoghurt samples were produced with bi-enzymatically modified as well as regular milk as outlined by Luzzi et al. (2020). The two multi-strain starter culture preparations YoFlex^®^ Premium 4.0 and ABT-100 from Chr. Hansen (Nienburg, Germany) were used for yoghurt production. According to the manufacturer, the traditional YoFlex^®^ Premium 4.0 yoghurt culture contained *S. thermophilus* and *Lb*. *delbrueckii* subsp. *bulgaricus* strains. The probiotic ABT-100, as indicated in the product data sheet, was comprised of *S. thermophilus*, *Lb. acidophilus* and a *Bifidobacterium* species, which was not specified to species level by the manufacturer (Chr. Hansen, Nienburg, Germany).

Three independent repetitions of each yoghurt production experiment were performed. Yoghurt samples were taken at four time points per experiment: immediately after the pasteurised yoghurt milk was inoculated with starter cultures, after fermentation (at the time point of cold storage when the yoghurt pH had reached 4.5) as well as after five and 21 days of storage at 4 °C. Sample sizes were 20 mL for inoculated milk and 20 g for yoghurt samples.

### Genomic DNA isolation

In preparation for DNA isolation, inoculated milk samples (20 mL) were centrifuged twice for 30 min at 6000×*g* at 10 °C in a Heraeus Multifuge (Thermo Fisher Scientific, Waltham, USA; Rotor: 75002005). The final supernatant (ca. 20 mL) from inoculated milk samples was stored at − 20 °C until further processing. Yoghurt samples (20 g) were mixed with 20 mL of 1 M Tris-HCl (pH 7.5; Carl Roth, Karlsruhe, Germany) and centrifuged twice for 20 min at 300×*g* at 10 °C in a Heraeus Multifuge. The final supernatant (ca. 40–45 mL) from yoghurt samples was also stored at − 20 °C until further analyses.

For further analyses, inoculated milk and yoghurt samples were thawed at room temperature. Bacterial cell lysis was performed using a lysis buffer comprised of 500 mM NaCl, 50 mM Tris–HCl (pH 8.0), 50 mM EDTA and 4% sodium dodecyl sulphate (buffer components from VWR International GmbH, Darmstadt, Germany). One volume of lysis buffer was added to all samples and these were mixed using an Intelli-Mixer RM-2M (ELMI, Calabasas, CA, USA) in the U2-mode at 80 rpm for 10 min. Following incubation in a 70 °C water bath (Julabo GmbH, Seelbach, Germany) for 20 min with shaking at 196 rpm, samples were centrifuged at 4 °C for 30 min at 6000×*g* in a Heraeus Multifuge. The fat layer that formed on top of the samples was removed with a sterile pipette tip and discarded, whilst the supernatant was transferred to a fresh 50 mL Falcon^®^ tube. This centrifugation step was repeated twice to obtain a clear lysate.

To each lysate tube, 10 M ammonium acetate (Merck, Darmstadt, Germany) amounting to 10% of the total sample volume was added. Samples were briefly mixed and incubated on ice for 20 min, before centrifuging at 4 °C for 30 min at 6000×*g*. One volume of 2-propanol (Carl Roth, Karlsruhe, Germany; pre-cooled to 4 °C) was added to the supernatant to precipitate the DNA. Samples were mixed and incubated on ice for 45 min, before centrifugation at 4 °C for 30 min at 6000×*g* and removal of the supernatant. Nucleic acid pellets were resuspended in 1 mL of 70% HPLC gradient grade ethanol (Carl Roth, Karlsruhe, Germany) and centrifuged at 13,000 rpm for 15 min at 4 °C in a Heraeus Fresco21 centrifuge (Thermo Fisher Scientific, Waltham, MA, USA), air-dried and resuspended in 200 µL 10 mM Tris–HCl (pH 8.0).

To each sample, 4 µL of DNAse-free RNase (10 mg/mL; VWR International GmbH, Darmstadt, Germany) was added and samples were incubated at 37 °C for 15 min. Thereafter, 30 µL of proteinase K solution (20 mg/mL; AppliChem GmbH, Darmstadt, Germany) and 200 µL of ‘Buffer AL’ from the QIAamp DNA Stool Mini Kit (QIAGEN GmbH, Hilden, Germany) were added to wash the sample and the mixture was incubated at 70 °C for 10 min. Two volumes of 99% HPLC gradient grade ethanol were added and samples were mixed thoroughly, prior to being transferred to the QIAamp Mini spin columns from the QIAamp DNA Stool Mini Kit (QIAGEN GmbH, Hilden, Germany) and centrifuged at 10,000 rpm for 1 min at room temperature. Subsequent washing steps with Buffers ‘AW1’ and ‘AW2’ were done with 500 µL of each wash buffer. Samples were eluted in 2 × 50 µL of pre-warmed ‘Buffer AE’ with centrifugation at 6000 rpm for 1 min to maximise the eluted DNA. The DNA concentration was measured using a Qubit^®^ 3.0 Fluorometer in conjunction with the Qubit™ dsDNA BR Assay Kit following the manufacturer’s protocol (Thermo Fisher Scientific, Darmstadt, Germany).

### Library preparation and sequencing

The bacterial community composition was determined using tag-encoded, MiSeq^™^ System-based 16S rRNA gene high throughput sequencing. The genomic DNA was diluted to 1.67 ng/µL prior to library preparation. Specific primers for the 16S rRNA gene V3 and V4 regions with Illumina adapter overhangs were used for amplification of the bacterial community in all samples (16S fw-meta 5′-TCG TCG GCA GCG TCA GAT GTG TAT AAG AGA CAG CCT ACG GGN GGC WGC AG-3′ and 16S rev-meta 5′-GCT TCG TGG GCT CGG AGA TGT GTA TAA GAG ACA GGA CTA CHV GGG TAT CTA ATC C-3′) (Klindworth et al. 2013). DNA amplification was carried out following the two-stage PCR protocol for 16S Metagenomic Sequencing Library Preparation provided by Illumina (Illumina Inc., San Diego, CA, USA). The size of the 16S rRNA gene PCR products was confirmed by means of automated electrophoresis using the Experion™ Automated Electrophoresis System in conjunction with the Experion™ DNA 12K Assay Kit according to the manufacturer’s instructions (Bio-Rad Laboratories, Inc., CA, USA). 16S rRNA gene sequencing was performed on a MiSeq^™^ System using the MiSeq Reagent Kit v3 following the manufacturer’s instructions (Illumina Inc., San Diego, CA, USA). Samples with a sequencing coverage of < 10K reads were re-sequenced and the reads from both runs were combined for subsequent analyses.

### Data analysis

The raw dataset was analysed using the de novo analysis pipeline of the IMNGS platform for ecology and diversity studies of the Technical University of Munich (Lagkouvardos et al. 2016). The raw fastq files were pre-processed using the Remultiplexor Perl script available through the IMNGS platform and then uploaded to the IMNGS pipeline, which implements the UPARSE algorithm from the USEARCH 8 (32-bit) package (Edgar 2010, 2013). The pipeline produced operational taxonomic unit (OTU) tables for each sample, which were uploaded to the SINA Arb Silva Aligner to compare these with both the Silva and rdp reference databases (Pruesse et al. 2012; Cole et al. 2014). The consolidated and verified OTU tables were used for statistical analysis using RStudio Version 1.1.423 by implementing the set of ‘Rhea scripts’ published by Lagkouvardos et al. (2017). These R scripts were used for 16S rRNA gene bioinformatic analysis of yoghurt samples by processing the OTU tables. Taxonomic binning to visualise the taxonomic composition of samples was performed in Microsoft Excel using the OTU tables generated by the Rhea pipeline. For statistical analysis, the Rhea pipeline applied a permutational multivariate analysis of variance (PERMANOVA) using distance matrices (vegan::adonis) to calculate the significance (*p*-values) of differences in microbial composition between groups of OTUs (Anderson 2017; Lagkouvardos et al. 2017). Three biological replicates were analysed for all sampling time points with the exception of the enhanced ABT-100 yoghurt sample after five days of storage at 4 °C, where only two biological replicates were analysed as no DNA could be recovered due to a methodological problem during DNA isolation in the third replicate.

## Results

In this study, 16S rRNA gene sequencing was used to analyse the microbial diversity of reformulated (sweetness-enhanced) yoghurt samples compared with regular yoghurt that was prepared using two different commercial mixed strain yoghurt starter culture preparations. Statistical analysis of samples taken during fermentation and post-acidification storage demonstrated that there was no significant difference between the microbial composition of yoghurt produced with either sweetness-enhanced or regular milk. This was observed for both the traditional YoFlex^®^ Premium 4.0 starter culture preparation containing *S*. *thermophilus* and *Lb*. *delbrueckii* subsp. *bulgaricus* (*p* = 0.401) strains and the mild-tasting, probiotic ABT-100 culture preparation containing *S. thermophilus*, *Lb. acidophilus* and a *Bifidobacterium* sp. strains (*p* = 0.657).

**Fig. 1:**
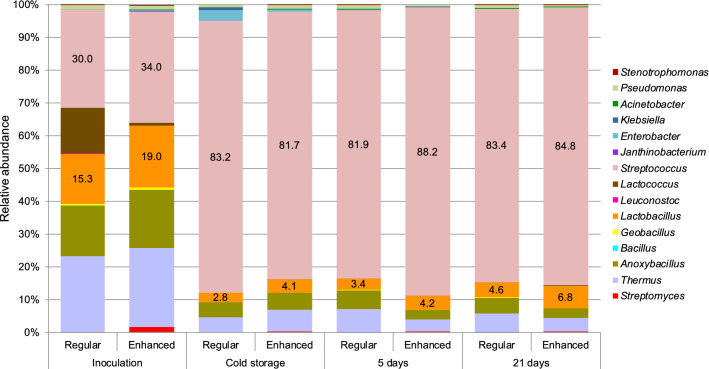
Relative microbial abundance (%) in yoghurt produced using regular and sweetness-enhanced (enhanced) milk using the traditional YoFlex^®^ Premium 4.0 starter culture. The relative abundance of the starter culture genera (*Streptococcus* and *Lactobacillus*) are indicated

In yoghurt samples produced with both the traditional and the probiotic yoghurt culture, the genera of lactic starter cultures dominated all samples by the end of the fermentation period at 43 °C. The diversity of microorganisms present in the yoghurt samples differed significantly (*p* = 0.001) between samples taken directly after milk inoculation with starter cultures and samples taken following fermentation, once the pH had reached 4.5 and the samples were cooled. In the traditional yoghurt culture (Fig. 1), the starter *Streptococcus* strain grew rapidly during fermentation, reaching a relatively dominant species abundance in all samples (81–88%) after fermentation. This was maintained during the 21-day storage period at 4 °C. The relative abundance of *Lactobacillus* DNA sequences, which presumptively stemmed from the starter *Lactobacillus* strain in these samples, was between ca. 2–7% of the total read abundances following fermentation.

**Fig. 2:**
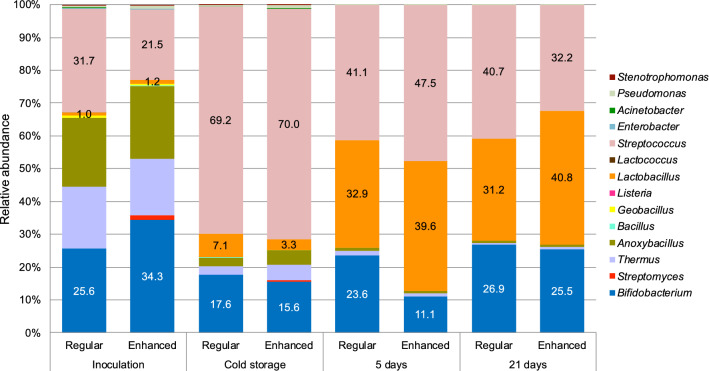
The relative abundance (%) of genera in yoghurt produced using sweetness-enhanced (enhanced) and regular milk using the probiotic ABT-100 culture. The relative abundances of the starter culture genera (*Streptococcus*, *Lactobacillus* and *Bifidobacterium*) are indicated

In yoghurt samples produced using the probiotic ABT-100 yoghurt starter culture preparation (Fig. 2), 16S rRNA metagenomic analyses confirmed the roughly equivalent inoculation levels of *Streptococcus* and *Bifidobacterium* [ca. 10^7^ cfu/mL for both these species, as demonstrated by Luzzi et al. (2020)], showing a ca. 20–35% relative abundance of each bacterial species. The *Lactobacillus* component of the mixed starter culture was inoculated about 1 log cfu/mL lower than *Streptococcus* and *Bifidobacterium*, and this was reflected by a relative bacterial abundance of only 1% in the metagenomic analysis of samples taken at inoculation. As a result of rapid growth of lactic acid bacteria during fermentation, an expected significant difference (*p* = 0.001) in microbial ecology, as determined by PERMANOVA analyses, was observed in samples taken before and after fermentation with the probiotic ABT-100 starter culture preparation. The abundance of bacteria of the *Streptococcus* genus, which presumptively stems from the starter culture, were measured at an abundance of ~ 70% after fermentation (at the time point of cold storage) in comparison to the those of the presumptive starter *Lactobacillus* (3–7%) and *Bifidobacterium* (15–17%) strains. Furthermore, the microbial diversity in the probiotic ABT-100 culture differed significantly (*p* = 0.001) between the beginning of cold storage and after five days of storage, presumptively due to the growth of the acidophilic starter *Lb. acidophilus*, further changing the relative abundances of all starter bacteria during storage. As post-acidification progressed, the relative abundance of probiotic *Lb. acidophilus* and *Bifidobacterium* species was observed to increase to 31–40% and 11–26%, respectively.

## Discussion

Sweetness-enhanced yoghurt samples taken during fermentation and post-acidification storage were analysed using 16S rRNA gene sequencing and statistical analyses demonstrated no significant difference between the microbial composition of this yoghurt compared to regular yoghurt. This held true for both yoghurt produced with the traditional YoFlex^®^ Premium 4.0 starter culture preparation as well as the mild-tasting, probiotic ABT-100 culture preparation. The implemented starter culture lactic acid bacteria therefore seemed to ferment the sweetness-enhanced milk in an equal manner to regular milk, despite the altered carbohydrate composition (Luzzi et al. 2020), which results in the presence of the monosaccharides glucose, galactose and fructose being more readily available for bacterial growth in the milk matrix.

The genera of lactic starter cultures predominated the microbial composition of all samples after fermentation of the sweetness-enhanced and regular milk (when yoghurt samples were placed in cold storage), showing a significant difference at this time point compared to the microbial diversity measured at inoculation. This change in microbial ecology between samples taken directly after milk inoculation with starter cultures and those taken following the fermentation period appears to confirm the essential growth of starter cultures needed for both acidification of the matrix and protection against the growth of unwanted organisms, not to mention to provide the desired product characteristics (Vedamuthu 2006).

In all traditional yoghurt samples, the presumptive starter *Streptococcus* strain grew rapidly during fermentation and their relative abundance clearly dominated over the abundance of DNA sequences from the presumptive *Lactobacillus* starter strains (Fig. 1). As high throughput sequencing cannot resolve bacterial taxonomy to the species level, the *Streptococcus* and *Lactobacillus* sequences obtained were thus termed presumptive *Streptococcus* and *Lactobacillus* starter strains in this study. As previously demonstrated by Luzzi et al. (2020) using traditional microbiological methods, the starter *Streptococcus* strain was inoculated nearly 3 log cfu/mL higher than the *Lactobacillus* strain in all yoghurt samples produced using this multiple-species traditional YoFlex^®^ Premium 4.0 starter culture preparation. The high relative abundance of *Streptococcus* DNA sequences observed in the product samples is likely to have overshadowed the relative abundance of *Lactobacillus* sequences detected, making the latter appear to be only minimally present after fermentation (Gobbetti et al. 2015). A similar effect could be observed at cold storage in samples produced using the probiotic ABT-100 yoghurt starter culture preparation (Fig. 2), where the abundance of *Streptococcus* strains clearly dominated the presumptive stater *Lactobacillus* and *Bifidobacterium* strains after fermentation. In this probiotic yoghurt, 16S rRNA metagenomic analyses confirmed the similar inoculation levels of *Streptococcus* and *Bifidobacterium* strains for both these species, as determined by Luzzi et al. (2020) for these samples using traditional microbiological methods.

The culture-dependent viable counts of the starter bacteria measured in a parallel study in the same reformulated yoghurt samples as analysed in this study showed a typical and expected microbial growth and acidification progression in the YoFlex^®^ Premium 4.0 starter culture for a traditional yoghurt starter culture (Luzzi et al. 2020). The ABT-100 culture displayed a decline in probiotic bacteria during storage, which is not uncommon in yoghurt, as a result of sensitivities of *Lb. acidophilus* and *Bifidobacterium* to the build-up of dissolved oxygen, organic acids and hydrogen peroxide produced by lactic acid bacterial metabolism (Gilliland and Speck 1977; Shah 2000; Ng et al. 2011). The results of the current 16S rRNA sequencing study therefore confirm the culture-dependent results presented previously by Luzzi et al. (2020).

The analysis of bacterial DNA from samples containing a diverse microbial population, such as can be found in fermented dairy products, does not allow for differentiation of DNA isolated from living, injured or dead cells. As the DNA from the entire yoghurt samples was isolated and sequenced in this study, it is impossible to distinguish the microbial abundance of live bacterial populations from dead cells in these samples. Hence, the increase in relative microbial abundance observed during storage of probiotic yoghurt produced with the ABT-100 culture, despite a decline in viable counts of *Lactobacillus* and *Bifidobacterium* species measured by Luzzi et al. (2020) in these samples, can be explained by the detection of DNA from the viable cells as well as from bacteria that were injured or died during yoghurt storage. In future studies, such confounding could be avoided by applying DNA-staining of dead or injured cells, for example using propidium monoazide or ethidium monoazide, and subsequent elimination of stained cells from the microbial sample, prior to DNA sequencing analysis (Rudi et al. 2005; Erkus et al. 2016).

The microbial diversity observed after inoculation of the milk for yoghurt production with both starter culture preparations displays a wide range of bacterial genera, which decreases after fermentation and during storage. This initial diversity probably stemmed from the microorganisms present in raw milk prior to milk pasteurisation and subsequent yoghurt manufacture. The microbiota of raw milk is known to be quite diverse, yet the pasteurisation process of milk prior to yoghurt production aims to inactivate the majority of unwanted spoilage and potentially pathogenic bacteria (Quigley et al. 2013). However, the DNA of these eliminated bacteria is still present in the milk and hence is also detected by the 16S rRNA metagenomics sequencing approach used in this study. Due to the rapid growth of starter culture bacteria during fermentation, the relative abundance of the DNA presumptively stemming from the starter culture bacteria dominated the samples at and after the time point of cold storage of the yoghurt after fermentation. Hence, the relative DNA abundances from non-living raw milk microbiota should not be strongly represented in relative abundance calculations as storage progresses. The quick dominance of starter lactic acid bacteria observed by the end of yoghurt fermentation in this study contributed to inhibiting the growth of any spoilage or potentially pathogenic bacteria, as shown using culture-dependent determinations by Luzzi et al. (2020), presumably through the creation of acidic conditions (Vedamuthu 2006).

In this study, a low abundance of *Pseudomonas* and other Proteobacteria (< 5%) was detected in all yoghurt samples produced with sweetness-enhanced and regular milk. Contrary to this, Zalewska et al. (2018) observed a corresponding high abundance of Firmicutes (e.g. *Lactobacillales*) but also of Proteobacteria (e.g. *Pseudomonadales*) in yoghurt sampled immediately after fermentation and an increase in Proteobacteria by the end of a 28-day storage period. However, the diverse microbial composition measured in (raw) milk samples, including *Lactobacillales* as well as *Pseudomonadales*, greatly depends on the farm and processing facility, as demonstrated in earlier studies by Quigley et al. (2013). Hence, comparison of microbial abundances between studies should only be made with caution. Furthermore, the opportunities for secondary contamination with *Pseudomonadales* after pasteurisation may be higher in a commercial milk processing environment than in pilot plant production as was the case in this study, as the milk holding tanks and processing equipment provide ample sources of contaminating bacteria.

In conclusion, the current study demonstrated that using sweetness-enhanced milk for yoghurt production did not affect the microbial ecology of the reformulated yoghurt samples in comparison to regular yoghurt samples. In both yoghurt samples produced using a traditional as well as a probiotic yoghurt starter culture preparation, the microbial biodiversity did not change between sweetness-enhanced and regular yoghurt at all sampling time points despite the altered carbohydrate composition of the milk matrix. From a microbiological perspective, this reformulation approach does not affect the essential growth of starter lactic acid bacteria needed to achieve the desired product characteristics of yoghurt, nor does it allow for a greater growth potential for spoilage or potentially pathogenic bacteria.

## Data Availability

The 16S rRNA gene sequencing raw data have been deposited in the Sequence Read Archive (SRA) with links to BioProject accession number PRJNA630675 in the NCBI BioProject database (https://www.ncbi.nlm.nih.gov/bioproject/).
